# Prognostic value of DAXX/ATRX loss of expression and ALT activation in PanNETs: is it time for clinical implementation?

**DOI:** 10.1136/gutjnl-2021-324664

**Published:** 2021-05-11

**Authors:** Ilaria Marinoni

**Affiliations:** Pathology, University of Bern, Bern, Switzerland

**Keywords:** pancreatic endocrine tumour, neuroendocrine cells, neuroendocrine tumors, pancreatic islet cell

Pancreatic neuroendocrine tumours (PanNETs) originate from the islets of Langerhans of the pancreas and they represent almost 3% of all pancreatic tumours. Over 50% of patients present nodal or distant metastasis at time of diagnosis, which results in an estimated 5-year survival in 27% of cases. On the contrary, localised well-differentiated PanNETs, especially those <2 cm, have a more indolent behaviour with 5 years survival of 93% of patients.^1^ Death domain-associated protein (*DAXX*) and or alpha-thalassemia/mental retardation X-linked chromatin remodeler (*ATRX*) are mutated in almost 40% of sporadic PanNETs, often in combination with *MEN1* mutations.^2^ *DAXX/ATRX* mutations result in loss of nuclear expression of the protein in the tumour tissue. DAXX and ATRX loss highly correlates with alternative lengthening telomeres (ALT) activation, although a causal role is still under investigation.^3^ Mechanisms driving PanNETs progression on DAXX and ATRX loss are still poorly understood. While the molecular mechanisms associated with DAXX and ATRX loss are still elusive, a clear role in PanNETs prognosis is emerging.^4–6^

In the present work, Hackeng *et al* consolidated the prognostic relevance of DAXX and ATRX loss in PanNETs, using a multicentre collective including 561 patients with PanNET.^6^ In multivariate analysis including tumour grade, lymph vascular invasion, perineural invasion, tumour stage, regional lymph node metastasis, loss of DAXX/ATRX expression and the presence of ALT, both loss of DAXX/ATRX and ALT activation were found independent prognostic factors for relapse free survival (p<0.001 for both).^6^

While, in non-metastatic PanNETs DAXX/ATRX loss and ALT activation clearly indicated a shorter disease free survival, in metastatic samples ALT activation seems to have an opposite role. Metastatic patient with ALT positive tumours showed a longer disease specific survival.^5 6^ Additionally, in previous studies, neither DAXX/ATRX loss nor ALT activation in primary tumours correlated with decreased overall patient survival.^4 5^ The emerging double role of ALT depending on the disease status of advance is intriguing; yet the reasons for this are still unknown.

Based on these evidences, there is a clear benefit of introducing DAXX/ATRX and/or ALT status in clinical routine for estimating relapse risk in PanNET patients in the absence of distant metastases.

Importantly, the authors were able to show an increased risk of relapse for DAXX/ATRX negative tumours also in the subgroup including 196 PanNET <2 cm.^6^ Indeed, despite their usual indolent behaviour, fewer than 15% of PanNETs ≤2 cm exhibit malignant features such as lymph node involvement, or recurrence after resection.^1^ Hence, management of PanNETs <2 cm represents a clinical dilaemma and markers able to predict tumour behaviour are needed. Although it is believed to be a late event in PanNETs progression and usually occurring in larger tumours, Hackeng *et al* found that a subset of <2 cm PanNETs (10%) already presents with DAXX and ATRX loss and ALT activation.^6^ PanNETs <2 cm with ALT activation and DAXX/ATRX loss have a significant shorter disease-free survival, suggesting that DAXX/ATRX status can provide an important biomarker for the management of localised small PanNETs.^6^

The islets of Langerhans include ive type of cells: α cells producing glucagon, β cells producing insulin, δ cells producing somatostin, ε cells producing ghrelin and PP secreting pancreatic polypeptide. Insulin and glucagon secreting cells represent the majority of the cells in the islets. Differentiation in the specific cell type is tightly regulated by the expression of cell lineage transcription factors. Aristaless-related homeobox (ARX) and PDX1 drive the differentiation of respectively, the α lineage and the β lineage. Recently, chromatin immunoprecipitation DNA-sequencing on H3K27ac super enhancer derived profiles, highlighted that PanNETs fall into two major subtypes, with epigenomes and transcriptomes that partially resemble islet α-cells and β-cells.^7^ The α-subtype is identifiable by ARX expression and the β subtype by PDX-1 expression; occasionally tumours may expressed both or none of the transcription factors. Alpha-subtype PanNET with ALT activation has a shorter disease free survival.^7^

Similarly, DNA methylation profiles are able to cluster PanNETs in three groups: α-like tumours positive for ARX, β-like tumours positive for PDX1 and intermediate tumours.^8^ Intermediate tumours remain positive for ARX in the majority of the cases and they are enriched for DAXX and ATRX mutant cases. While the α-like and intermediate PanNET include mainly non functioning tumours, the β-like include, as expected, insulinomas.^8^ Despite enrichment of DAXX/ATRX mutant tumours, high grade and large tumour size in the α like group, ARX and PDX1 expression per se are not able to stratify patients with different risk of relapse.^6 8^ Indeed, the authors showed that ARX and PDX1 expression was equally distributed in primary tumours and metastasis.^6^ Therefore, rather than prognostic markers ARX and PDX1 may instead indicate the tumour cell of origin. Interestingly, enrichment of DAXX/ATRX loss and *MEN1* mutations in tumours positive for ARX suggest that α cells are more susceptible to these mutations while β cells have a peculiar mutations spectrum including *YY1*, pointing out the difference between non functioning PanNET and insulinoma.^9^ Only malignant insulinoma showed positivity for ARX and ALT, suggesting a similar development to non-functioning tumours.^10^

In conclusion, DAXX and ATRX expression and ALT activation should be included as prognostic factor for localised NF-PanNETs to identify those patients at higher risk of relapse. This patient population would also be relevant for future adjuvant therapy trials. In PanNET <2 cm loss of DAXX/ATRX or ALT should be an indication for surgery. Lastly, given the fact that DAXX/ATRX loss is not observed in NET from other origin, except a small fraction of lung NET, it may be a useful biomarker for the identification of primary tumours in the setting of metastasis of unknown origin.^6^

However, the emerging different role of ALT activation and DAXX/ATRX loss in metastasised tumours and in affecting patient overall survival poses some questions for its clinical implication. The reasons for this different behaviour are not clear yet, additional mutations may take over during the progression or different response to treatments may play a role. Indeed, ALT positive tumours showed highcopy number variation (CNV), which may results in a more sensitivity to specific therapies. Additional investigations in this respect are crucial to identify the potential Achille’s heels of these tumours. While the role of DAXX/ATRX loss for prognosis is now wildly recognised, it is still to clarify if it could a have a predictive function as well. Currently, in fact, there is no way to select a specific therapy for this subtype of tumours; more studies in this direction are essential.
